# Disorganization of neocortical lamination in focal cortical dysplasia is brain-region dependent: evidence from layer-specific marker expression

**DOI:** 10.1186/2051-5960-1-47

**Published:** 2013-08-08

**Authors:** Susanne Fauser, Ute Häussler, Catharina Donkels, Susanne Huber, Julia Nakagawa, Marco Prinz, Andreas Schulze-Bonhage, Josef Zentner, Carola A Haas

**Affiliations:** 1 Experimental Epilepsy Research, Department of Neurosurgery, University of Freiburg, Breisacherstr. 64, Freiburg 79106, Germany; 2 Epilepsy Center, Department of Neurosurgery, University of Freiburg, Freiburg, Germany; 3 Institute of Neuropathology, University of Freiburg, Freiburg, Germany; 4 BIOSS Centre for Biological Signaling Studies, University of Freiburg, Freiburg, Germany; 5 Department of Neurosurgery, University of Freiburg, Freiburg, Germany; 6 Bernstein Center Freiburg, University of Freiburg, Freiburg, Germany; 7 Faculty of Biology, University of Freiburg, Freiburg, Germany; 8 University of Ulm, Department of Neurology, Ulm, Germany

**Keywords:** Development, Epilepsy, Migration, Cytoarchitecture, Layer-specific markers, Interneuron, Neocortex

## Abstract

**Background:**

Focal cortical dysplasias (FCD) are local disturbances of neocortical architecture and a common cause of pharmaco-resistant focal epilepsy. Little is known about the pathomechanisms leading to architectural abnormalities associated with FCD.

**Results:**

In the present study we compared 52 FCD cases originating from the frontal or temporal lobe with or without Ammon’s horn sclerosis (AHS) with regard to structural and molecular differences. We applied layer-specific (ER81, RORß, SMI32, TLE4) and interneuron (calbindin, parvalbumin) markers by means of immunohistochemistry, *in situ* hybridization (ISH), and real time RT-PCR and correlated our findings with clinical parameters. We found that: (1) Structural abnormalities were most prominent in layers III-VI including changed morphology of individual neurons or dispersion, blurring and thinning of layers. These alterations were most pronounced in isolated frontal FCD, whereas the most homogeneous group was FCD IIIa. (2) Numbers of calbindin- and parvalbumin-positive interneurons varied considerably within the different FCD groups, but were not generally reduced. A significant decrease was only found for calbindin-positive interneurons in frontal FCD, and for parvalbumin-positive interneurons in FCD IIIa. (3) Interestingly, FCD IIIa presented with significant changes in the numbers of calbindin- or TLE4-positive neurons when compared to isolated FCD or controls. (4) Correlations between clinical and cellular parameters strongly depended on FCD localisation and age of the patients.

**Conclusions:**

In summary, our data suggest that late cortical development is disturbed in FCD, yet most likely by different causes depending on brain region, FCD type and FCD severity.

## Background

Malformations of cortical development, in particular focal cortical dysplasia (FCD), are frequent causes of medically intractable focal epilepsy. FCD are increasingly recognized in epilepsy patients owing to improved imaging techniques [1-6] and have been identified in 20-25% of patients with focal epilepsy [7]. FCD is thought to result from disturbances during pre- and perinatal brain development which is traditionally divided into three stages: proliferation, migration and cortical organisation [8]. Although FCD is a common cause of epilepsy, there is sparse knowledge about the underlying pathomechanisms. FCD presents with variable histological findings pointing to different pathogenetic mechanisms, also reflected by several classifications [9-12]. Until recently, FCDs were mainly distinguished by the presence or absence of Balloon cells. Although the histological abnormalities in FCD with Balloon cells are more prominent than in FCD without Balloon cells, there is no difference regarding the severity of epilepsy [11,13-15]. In both patient groups a high seizure frequency and a pharmaco-resistant course of the disease is common [16-18]. A further topic of ongoing debate is whether temporal lobe dysplasia associated with Ammon’s horn sclerosis (AHS) is morphologically and pathogenetically different from temporal lobe dysplasia without AHS. The recent FCD classification system proposed by the Task Force of the International League Against Epilepsy (ILAE) Diagnostic Commission [19] limits type I FCD to isolated lesions and introduces type III FCD for architectural dysplasia in combination with other principal lesions (type IIIa when associated with AHS) on the basis that the isolated forms may evolve during cortical development, whereas the others may be acquired as a result of the main epileptogenic lesion (i.e., AHS).

A detailed characterization of architectural disturbances has recently become possible by using layer-specific markers which specifically label subsets of cortical neuron types with restricted laminar distribution [20-22]. There is strong evidence that neuron identity, neuron type and laminar fate are already specified, before the onset of migration towards the pial surface [23,24]. Once specified, the neuron type identity and laminar fate are determined, even if cell migration is abnormal [21,25]. In addition to migration failure abnormal specification or differentiation of the neuron type or laminar fate can contribute to cortical disorganisation [26-28]. Over the last few years several studies have used layer-specific markers to learn more about the cytoarchitectural disturbances in FCD and other structural brain defects such as subcortical nodular heterotopia [22,29-31]. All these studies focused either on the temporal lobe or examined only small patient cohorts.

In the present study we investigated a large cohort of FCD specimens originating from the frontal or temporal lobe with and without AHS with the aim to gain new insights in the pathology of dyslamination using layer-specific and interneuron markers. We present evidence that cytoarchitectural abnormalities in isolated FCD are brain region-dependent and that the presence of AHS determines a more homogeneous FCD entity when compared to isolated FCD types.

## Results

### Expression of layer-specific and interneuron markers in control cortex

**Figure 1:**
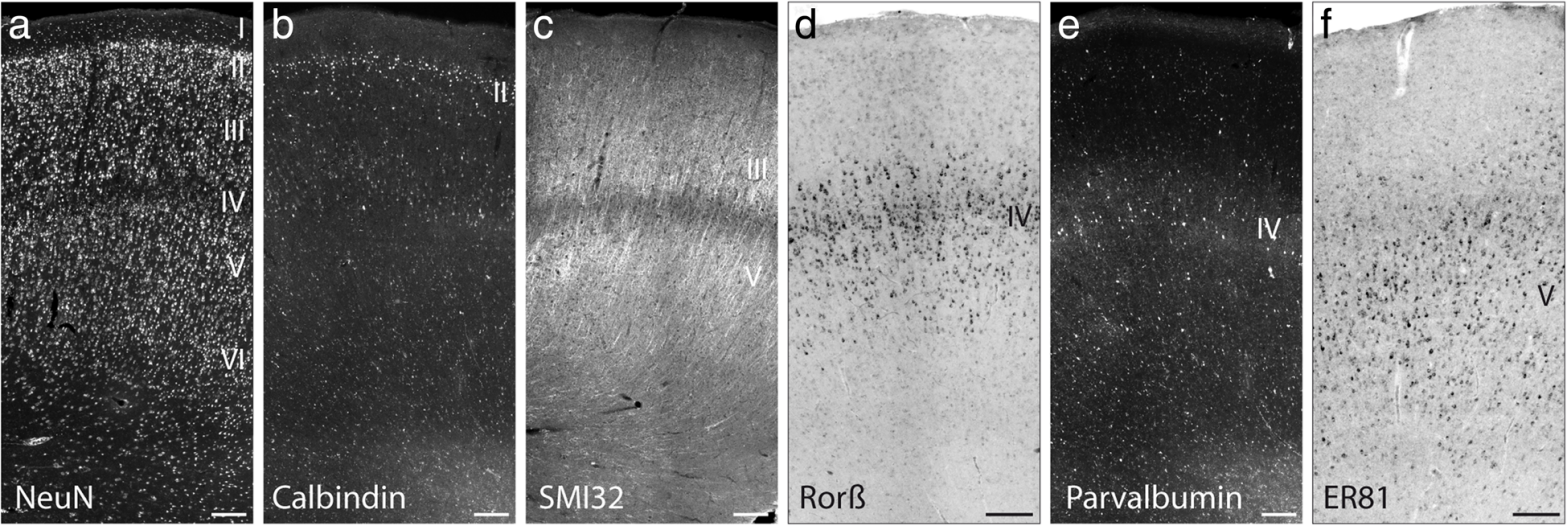
**Expression patterns of layer-specific markers in normal human temporal cortex.** Adjacent tissue sections were either immunolabeled for NeuN, all layers **(a)**, calbindin, mainly layer II **(b)**, SMI32, layers III and V **(c)** and parvalbumin, mainly layer IV **(e)** or processed for ISH for RORß, layer IV **(d)**, and ER81, layer V **(f)** mRNAs. **(a)** NeuN staining shows the six neocortical layers indicated by Roman numerals. **(b)** Calbindin-positive interneurons are mainly located in layer II and to a lesser extent in layer III-IV **(c)** SMI32-positive neurons are located in layer III and layer V/VI. There is always a clear demarcation of layer IV in terms of a SMI32-free area between layer III and V. The apical dendrites of SMI32-positive neurons in layer III have a parallel orientation and reach the lower border of layer I. **(d)** The typical appearance of RORß mRNA-positive neurons is a dense band on the level of layer IV, organised in small columns and with an extension of less dense RORß-positive “branches” into the neighbouring layers III and V. **(e)** Parvalbumin-positive interneurons are mainly seen in layer IV of the human neocortex. At this level they form a well visible fiber plexus which refers to the parvalbumin staining in neurites and parvalbumin-containing synaptic terminals. **(f)** Strongly stained ER81 mRNA-expressing neurons are always seen in an infra-granular position. In the subcortical white matter, however, no abundance of ER81-positive neurons is observed in the control group. Additionally, a faint and variable ER81-staining is seen in pyramidal neurons of layer III. Scale bars: 250 μm.

To visualize the six-layered structure of normal human neocortex, immunolabeling or ISH for the pan-neuronal marker NeuN, for layer-specific markers (SMI32, RORß, ER81 and TLE4) and for subpopulations of interneurons (calbindin, parvalbumin) were carried out on control specimens from the frontal and temporal lobe (Figure 1). The six cortical layers - visualized by NeuN immunohistochemistry - were well distinguishable (Figure 1a). Calbindin, a marker for double-bouquet, Martinotti and neurogliaform interneurons in layer II and to a lesser extent in layer III-IV [32], stained interneurons with a distinct maximum at layer II (Figure 1b). Anti-SMI32, which recognizes non-phosphorylated neurofilament H, was used as marker for cortico-spinal projection neurons in layer III and layer V/VI [21,33]. In the control cortex, SMI32-positive neurons were located in layer III and layer V/VI. There was always a clear demarcation of layer IV in terms of a SMI32-free area between layer III and V. The apical dendrites of SMI32-positive neurons in layer III had a parallel orientation and reached the lower border of layer I (Figure 1c). RORß was used as a marker for internal granule cell layer neurons [21,34]. In controls, the typical appearance of RORß mRNA-positive neurons was a dense band on the level of layer IV, organised in small columns and with an extension of less dense RORß-positive “branches” into the neighbouring layers III and V (Figure 1d). Parvalbumin-positive interneurons were mainly located in layer IV, where they formed a well visible axonal plexus, containing immunolabeled neurites and synaptic terminals (Figure 1e). To a lesser extent parvalbumin-positive neurons were located in layers III and V in agreement with previous studies [35]. ER81 mRNA is expressed in cortico-spinal and cortico-cortical projection neurons in layer V and VI [21,36]. Concomitantly, in our controls, strongly stained ER81 mRNA-expressing neurons were always seen in an infra-granular position (below layer IV). Additionally, a faint and variable ER81 mRNA signal was seen in pyramidal neurons of layer III (Figure 1f). TLE4 is a transcriptional activator specifically localized in nuclei of layer V/VI neurons [21]. In all control groups, TLE4-positive neurons were always visualized in an infra-granular position (data not shown).

### Analysis of lamination in isolated frontal and temporal FCD and in FCD with AHS

With the aim to investigate potential changes in cortical layering in isolated FCD cases from the frontal (FCx) and temporal (TCx) lobe and temporal FCD associated with AHS (FCD IIIa) we performed immunocytochemistry for NeuN, SMI32 and TLE4 and ISH for RORß and ER81 in serial sections of all cases destined for morphological analyses (Additional file 1: Table S1).

**Figure 2:**
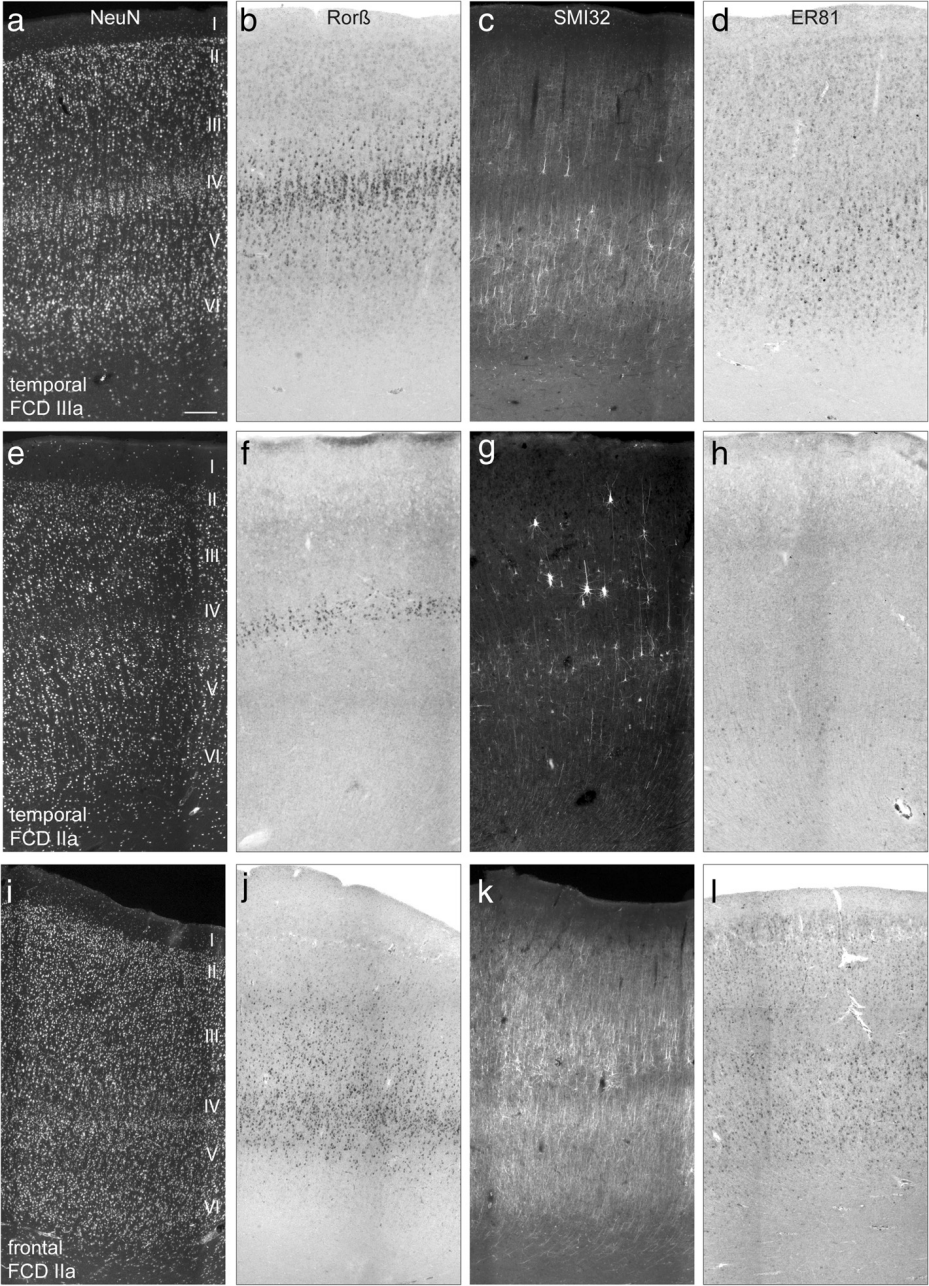
**Expression patterns of layer-specific markers in FCD cases originating from the temporal or frontal lobe. (a**-**d)** FCD IIIa; temporal. **(e**-**h)** FCD IIa, temporal; **(i**-**l)** FCD IIa, frontal. Representative micrographs of NeuN- **(a**,**e**,**i)** and SMI32-immunostained **(c**,**g**,**k)** sections or ISH for RORß **(b**,**f**,**j)** and ER81 **(d**,**h**,**l)** mRNAs are shown. Scale bar in a (valid for all micrographs): 250 μm. **(a**-**d)**. FCD III; a temporal. **(a)** In this patient a clear hexalamination with strong columnisation is discernible. **(b)** Layer IV contains a dense band of RORß-positive neurons with columnar distribution. **(c)** Density of SMI32-positive neurons appears reduced in layer III. **(d)** Abundance of ER81 mRNA-positive neurons is similar to the control. **(e**-**h)** FCD IIa, temporal; **(e)** In this patient, NeuN staining shows a “hypoplastic” layer IV and a blurred transition between layer V and VI. **(f)** A slim layer IV with only a few RORß mRNA-positive neurons is present. RORß-positive “branches” in layer III and V as seen in **(b)** are not detectable. **(g)** SMI32-positive neurons appear severely reduced. In layer III several large and dysmorphic SMI32-positive neurons are visible. Note that SMI-positive neurons have a parallel orientation of their apical dendrites and are in a correct position sparing layer IV. **(h)** Strong rarefication of ER81 mRNA expressing neurons in layer V. **(i-l)** FCD IIa, frontal. **(i)** Note a severely disturbed laminar organization with a gradient from right to left. Several pyramidal cells have an abnormal large size. **(j)** RORß mRNA-positive neurons are dispersed without columnar arrangement. **(k)** SMI32-immunostaining shows mainly a bilaminar distribution, which is disturbed in the left portion of the section where SMI32-positive neurons seem to “invade” layer IV **(l)**. ER81 mRNA-positive neurons are abundant in layer V.

NeuN immunolabeling revealed that layers I and II appeared basically unchanged in all FCD groups, whereas several abnormalities were observed in layers III - VI (Figure 2): In some cases a clear demarcation of layer IV was missing (Figure 2i, left half) and a blurred border between layer V and VI was a common finding (Figure 2 e,i).

**Figure 3:**
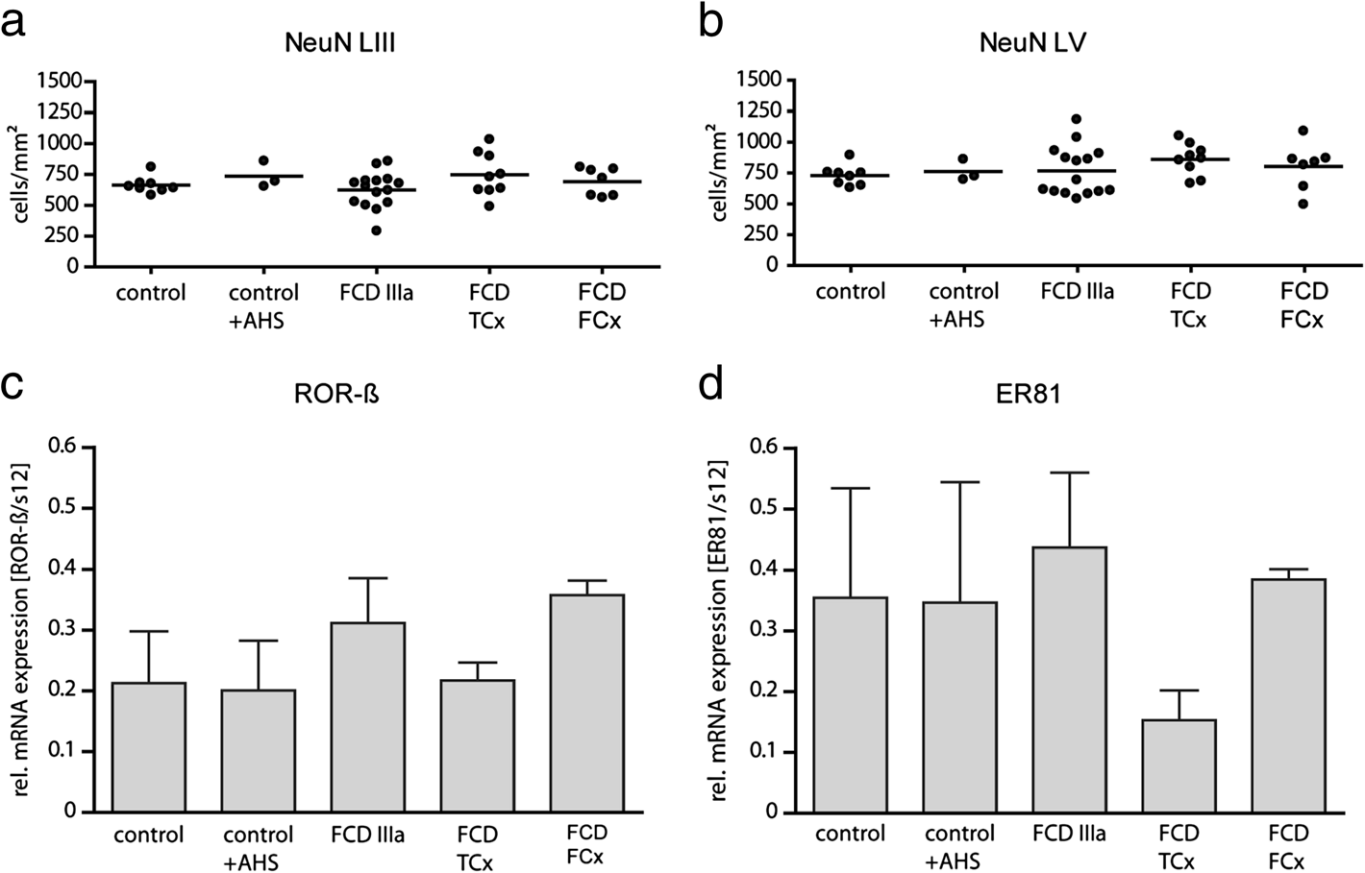
**Quantitative evaluation of neuron densities and layer-specific gene expression in controls and FCD cases. (a**,**b)** Quantification of NeuN-positive neurons in layers III **(a)** and V **(b)** of control cases and different types of FCD. NeuN immunolabeled neurons were counted as described in Material and Methods. Note that cell density (number of cells/mm^2^) did not differ between controls or FCD cases (one-way ANOVA). **(c**,**d)** Quantification of RORß **(c)** or ER81 **(d)** mRNA expression by real time RT-PCR. (control: n = 4; control + AHS: n = 3; FCD IIIa: n = 13; FCD TCx: n = 3; FCD FCx: n = 4). **(c)** Expression levels of RORß mRNA were similar in all groups (one-way ANOVA). **(d)** Expression levels of ER81 mRNA were comparable between controls and FCD cases (one-way ANOVA). TCx, temporal cortex; FCx, frontal cortex.

However, when the density of NeuN-positive cells was determined in layer III [cells/mm^2^ given as mean ± standard error of the mean (SEM): control 665 ± 24 (n = 8), control + AHS 737 ± 63 (n = 3), FCD IIIa 625 ± 38 (n = 15), FCD TCx 749 ± 59 (n = 9), FCD FCx 692 ± 42 (n = 7)] and layer V [control 730 ± 29 (n = 8), control + AHS 763 ± 51 (n = 3), FCD IIIa 767 ± 51 (n = 15), FCD TCx 862 ± 43 (n = 9), FCD FCx 804 ± 71 (n = 7)], we did not find any significant differences in cell numbers, neither between FCD groups nor between patients and controls (Figure 3a,b).

In the following we will describe the abnormalities found in individual layers in the three different patient groups using layer-specific markers.

Immunostaining for SMI32 revealed that the apical dendrites of pyramidal neurons in layer III appeared shortened and pyramidal neurons of layer V/VI did not respect the white matter border. This pattern occurred only in temporal FCD cases with and without AHS and was observed in around 50% of these patients (Figure 2c,g). In addition, large and misshaped SMI32-positive neurons were found in layers III and V/VI (Figure 2c,g).

Frontal FCD, however, showed additional abnormal features: SMI32-positive pyramidal cells appeared (i) denser especially in the outer portion of layer III (2 cases), (ii) were displaced into layer IV (5 cases) or (iii) were mal-orientated and/or hypertrophic with distorted apical dendrites in layers III and V (3 cases) (Figure 2k).

ISH for RORß mRNA showed that in temporal FCD the columnar organisation of RORß mRNA-positive neurons was basically preserved (Figure 2b) indicating that layer IV was not disturbed. Only in one case, a strong rarefication of RORß mRNA-expressing neurons in layer IV was observed (Figure 2f). In frontal FCD cases a more heterogeneous picture emerged including dispersion into layer III (3 cases) (Figure 2j) or rarefication (2 cases) of RORß mRNA-positive cells.

Next, we performed ISH for ER81 mRNA to investigate layers V and VI. In all FCD cases ER81 mRNA expressing neurons were mainly located in an infra-granular (below layer IV) position, but in contrast to controls, they were less densely packed and in some cases strongly reduced. In addition, ER81 mRNA expressing cells were detected in the underlying white matter (Figure 2d,l). Only in a few cases with isolated temporal FCD the distribution of ER81 expression was restricted to layer V/VI as seen in controls (Figure 2h).

**Figure 4:**
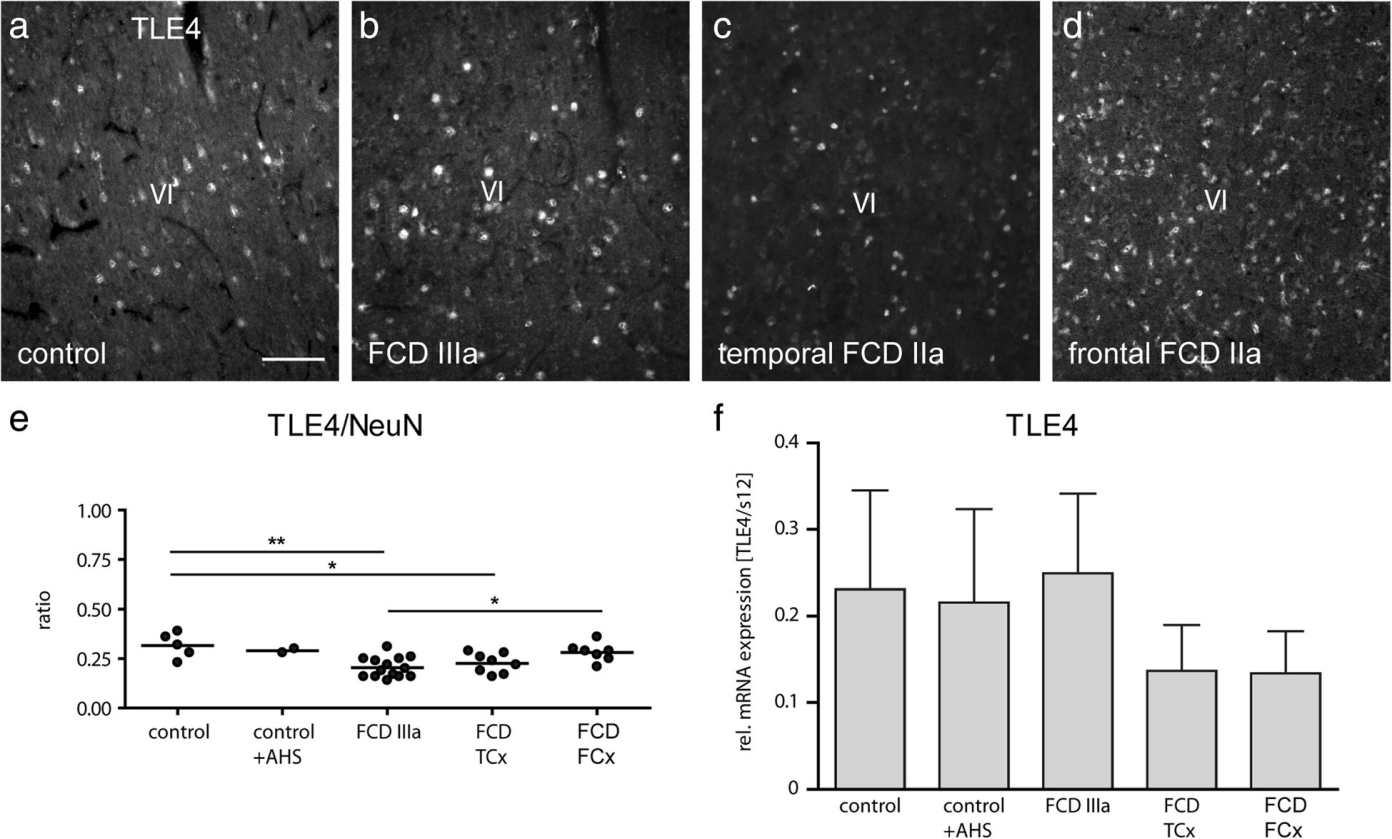
**TLE4 expression in controls and FCD cases originating from the temporal or frontal lobe. (a**-**d)** Representative sections of TLE immunostaining in control cortex **(a)**, FCD IIIa **(b)**, FCD IIa temporal **(c)** and FCD IIa frontal **(d)**. Scale bar: 100 μm. TLE4-positive nuclei are present in layer VI of all subgroups indicating the preservation of layer VI. **(e)** Ratio of TLE4/NeuN cell densities in controls and FCD cases. Numbers of TLE4-positive cells are significantly reduced in FCD IIIa versus control (p < 0.01, one-way ANOVA with Tukey’s post-test) and to FCD FCx (p < 0.05) and in FCD TCx versus control (p < 0.05). **(f)** Quantification of TLE4 mRNA expression levels by real time RT-PCR. TLE4 mRNA expression did not significantly differ between control or FCD cases.

Finally, we used immunolabeling for TLE4 to characterize potential changes in layer V/VI. Like in controls, TLE4-positive nuclei were always located in an infra-granular position in FCD patients (Figure 4a-c). Cell counting revealed that patients with FCD IIIa and isolated temporal FCD showed a significantly lower ratio of TLE4-positive cells relative to NeuN-positive cells when compared to controls. In FCD IIIa the number was also significantly reduced compared to frontal FCD (Figure 4e). Assuming that the number of NeuN-positive cells remains constant (as shown above) this indicates a relative reduction of TLE4-expressing cells among all neurons [TLE4/NeuN: control 0.32 ± 0.03 (n = 5), control + AHS 0.29 ± 0.01 (n = 2), FCD IIIa 0.21 ± 0.01 (n = 14), FCD TCx 0.23 ± 0.02 (n = 8), FCD FCx 0.28 ± 0.02 (n = 7)].

Taken together, we found that structural alterations mostly affected layers III-VI including changed morphology of individual neurons, and dispersion, blurring and thinning of layers. These alterations were most pronounced and heterogeneous in isolated frontal FCD, whereas the most homogeneous group was FCD IIIa.

In order to quantify the expression differences of RORß and ER81 mRNA observed on the morphological level, we performed real time RT-PCR with another cohort of patients including the same FCD types and controls (for details see Additional file 1: Table S2). We found no significant differences of RORß or ER81 mRNA expression between the FCD groups and controls (Figure 3c) most likely due to the high variability within individual groups.

### Quantification of interneurons

We complemented our characterization of laminar abnormalities in FCD with the investigation of calcium-binding proteins, parvalbumin and calbindin, known to be expressed in different subpopulations of interneurons.

**Figure 5:**
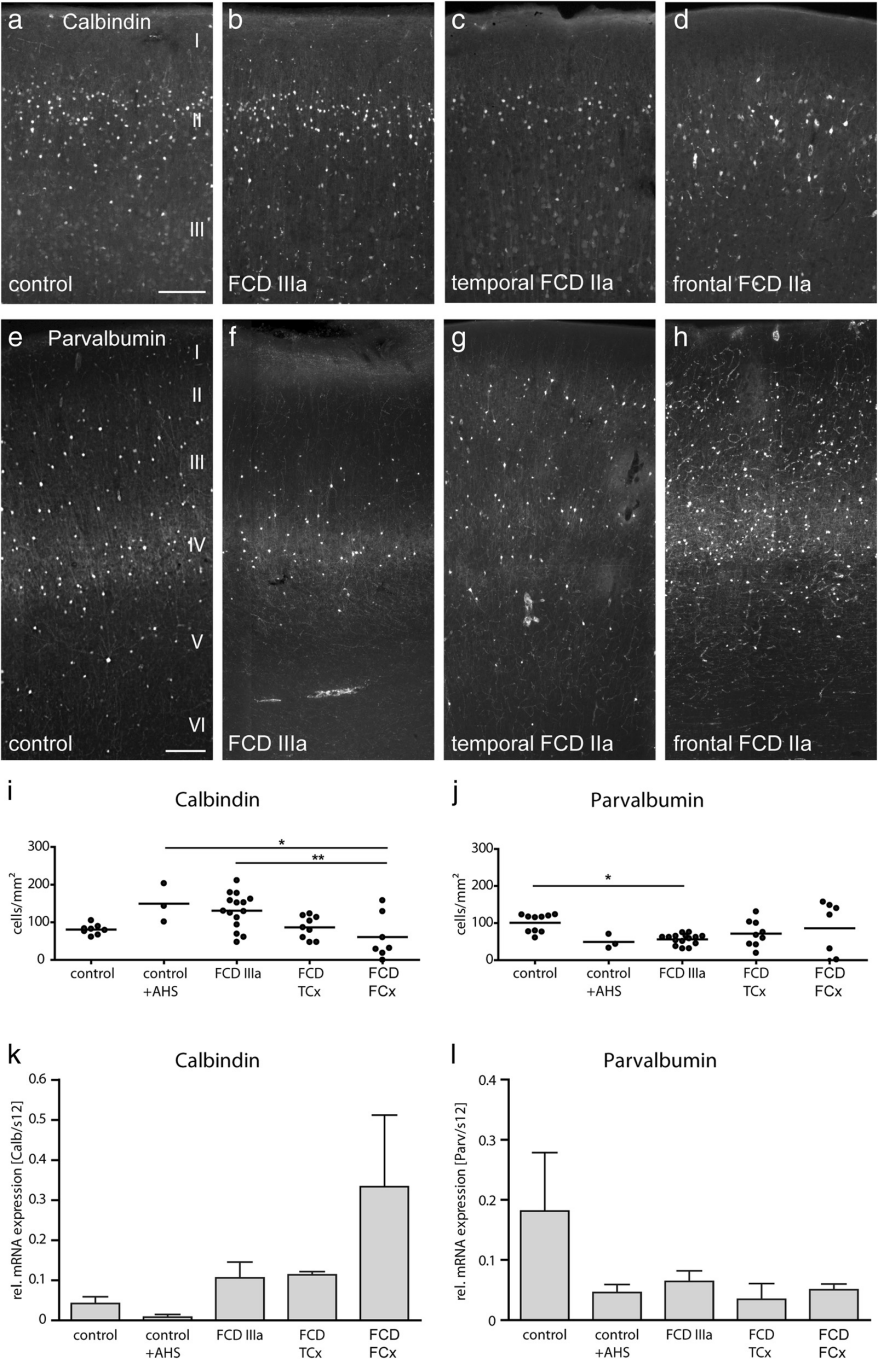
**Distribution of calbindin- and parvalbumin-positive interneurons in controls and FCD cases from the temporal or frontal lobe. (a**-**h)** Representative sections of calbindin **(a**-**d)** and parvalbumin immunostainings **(e**-**h)** in control cortex **(a**,**e)**, FCD IIIa **(b**,**f)**, FCD IIa temporal **(c**,**g)** and FCD IIa, frontal **(d**,**h)**. Scale bars: 200 μm **(a**-**d)**; 250 μm **(e**-**h)**. **(a)** Control. A dense ribbon of calbindin-positive neurons can be observed at the level of layer II. **(b)** FCD IIIa. Calbindin-positive neurons are similarly distributed when compared to control. **(c)** FCD IIa, temporal. Density of calbindin-positive neurons appears reduced in layer II. **(d)** FCD IIa, frontal. Reduction of calbindin-positive neurons, individual cells show a dysmorphic appearance. **(e)** Control. Parvalbumin-positive cells and a dense axonal plexus are concentrated at layer IV, but are also seen in layer III and V. **(f)** FCD IIIa. In this patient the axonal plexus is smaller and cell numbers are reduced, especially in layer III. **(g)** FCD IIa, temporal. Note the rudimentary and discontinuous axonal plexus in layer IV, but an accumulation of parvalbumin-positive cells in layer II. **(h)** FCD IIa, frontal. Parvalbumin-positive neurons appear more numerous and are widely distributed over the layers II-IV. **(i**,**j)**. Densities of calbindin- **(i)** and parvalbumin-positive (layer IV) neurons **(j)** in controls and FCD cases. **(i)** Densities of calbindin-positive cells were significantly reduced in FCD FCx when compared to AHS groups (control + AHS, p < 0.05, FCD IIIa, p < 0.01. One-way ANOVA with Tukey’s post-test). **(j)** Densities of parvalbumin-positive cells were significantly reduced in FCD IIIa when compared to controls (p < 0.05). All other FCD groups did not differ significantly. **(k**,**l)** Quantification of calbindin and parvalbumin mRNA expression does not show significant differences between controls or FCD cases.

Calbindin-positive interneurons were located mainly in layer II in controls and in all FCD groups (Figure 5a-d). In some cases, large dysmorphic calbindin-positive neurons were observed in layer II (Figure 5d). Cell counting revealed variability in the density of calbindin-positive neurons in FCD compared to controls [control 80 ± 5 (n = 8), control + AHS 150 ± 29 (n = 3), FCD IIIa 130 ± 12 (n = 15), FCD TCx 87 ± 10 (n = 9), FCD FCx 61 ± 23 (n = 7)]. The highest variability was found in frontal FCD (Figure 5i). Interestingly, mean numbers of calbindin-positive cells were higher in the groups with AHS (control + AHS, FCD IIIa) than in all other groups. These differences turned out to be significant when compared to frontal FCD (Figure 5i). Real-time RT-PCR did not detect any significant differences of calbindin mRNA levels in controls and all FCD groups (Figure 5k).

Immunolabeling for parvalbumin showed a high density of parvalbumin-positive cell bodies and a well distinguishable axonal plexus in layer IV in all FCD groups and controls (Figure 5e-h). In some cases parvalbumin-labeled neurons were scattered in layer II/III (Figure 5e-h). When cell densities were determined by cell counting in layer IV [control 101 ± 8 (n = 10), control + AHS 49 ± 11 (n = 3), FCD IIIa 56 ± 4 (n = 15), FCD TCx 72 ± 12 (n = 9), FCD FCx 86 ± 27 (n = 7)], a significant reduction of parvalbumin-positive cells in FCD IIIa was found compared to controls. All other groups did not differ significantly. Cell numbers were most variable in frontal FCD, but were more homogeneous within the other groups than in the calbindin-positive interneuron population (Figure 5j). No significant differences of parvalbumin mRNA levels were found between all groups (Figure 5l).

### Correlation with clinical parameters

**Figure 6:**
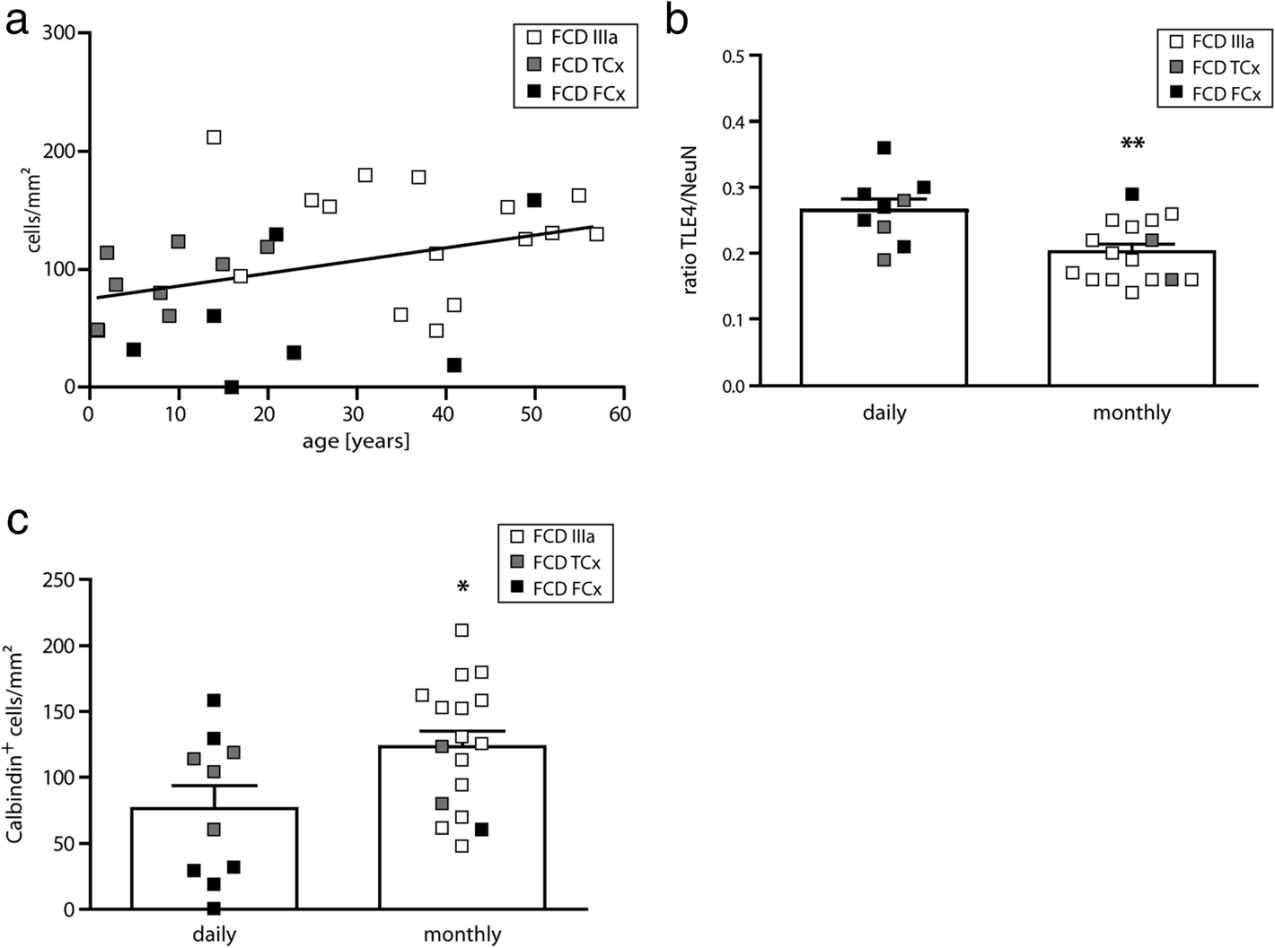
**Correlation of cellular and clinical parameters. (a)** Correlation between age at surgery and the density of calbindin-positive cells in isolated frontal and temporal FCD and FCD IIIa. We found a significant correlation (r^2^ = 0.13) of lower density of calbindin-positive cells with lower age at surgery. **(b)** Ratio of TLE4/NeuN positive cells for patients with daily or monthly seizures. Patients with monthly seizures had a significantly lower ratio of TLE4-positive cells (Student’s t-test, p = 0.004). Please note that most FCD IIIa cases are in the group with monthly seizures whereas isolated FCD cases have daily seizures. **(c)** Density of calbindin-positive cells in patients with daily or monthly seizures. Patients with daily seizures have significantly less calbindin-positive cells (Student’s t-test, p = 0.029). Again, note the distribution of the different FCD cases within the two seizure groups.

The parameters (1) age at epilepsy onset, (2) age at surgery, (3) duration of epilepsy and (4) seizure frequency were correlated with densities of interneurons and pyramidal cells. We could not find any correlation between age at epilepsy onset or duration of epilepsy and cell densities. However, we found a significant correlation between age at surgery and the density of calbindin-positive interneurons: lower numbers of calbindin-positive cells were correlated with low age at surgery (linear regression, r^2^ = 0.13, Figure 6a). Notably, most patients with a high number of calbindin-positive cells and a higher age at surgery belonged to the FCD IIIa group, while patients with isolated FCDs were younger at surgery and had lower numbers of calbindin-positive cells, matching the results described above. All other cellular parameters did not show any correlation with clinical parameters; in particular, there was no correlation between epilepsy duration and neuronal cell counts in the pyramidal cell layers.

Concerning seizure frequency, statistically significant differences were seen in the ratio of TLE4/NeuN-positive cells between patients with daily seizures and patients with only monthly seizures: the ratio was higher in patients with higher seizure frequency (Student’s t-test, p = 0.004; Figure 6b). Furthermore, the number of calbindin-positive neurons was significantly higher in patients with monthly seizures than in patients with daily seizures (p = 0.029; Figure 6c). However, these results may be biased by a localization- and age-dependence of seizure frequency: we found that patients with frontal FCD had mostly daily seizures (6 out of 7 patients), patients with isolated temporal FCD had daily or monthly seizures (4/9 daily, 2/9 monthly, 3 unknown) and patients with FCD IIIa had mainly monthly seizures (14 of 15 patients, 1 unknown).

## Discussion

The main findings of our study are the following: (1) Structural abnormalities mainly affected layers III-VI and to a lesser extent layer II, including altered morphology of individual neurons or blurring or thinning of layers. These alterations were most pronounced in isolated frontal FCD, whereas the most homogeneous group was FCD IIIa. (2) Numbers of calbindin- and parvalbumin-positive interneurons varied considerably, but were not generally reduced in isolated FCD. (3) Interestingly, FCD IIIa presented with increased numbers of calbindin-positive neurons when compared to frontal FCD, but a loss of parvalbumin-positive neurons when compared to controls. FCD IIIa and isolated temporal FCD showed decreased numbers of TLE4-positive neurons when compared to controls. (4) Correlations between cellular and clinical parameters were highly dependent on FCD localization and age of the patient.

### Basic preservation of cortical lamination

In the present study we show by application of several layer-specific markers that isolated FCD (Ia, Ib and IIa) in the temporal and frontal lobe and FCD IIIa are characterized by the principal maintenance of layering. In all cases, individual layers were either dispersed or blurred, but a complete disorganization was never observed. The most severe alterations were seen in layers III-VI, while layer II was affected to a lesser extent and only in some patients. These observations are basically in agreement with a recent study [30] in which, however, only a small number of temporal FCD cases was investigated. In contrast, the present study included a large cohort of FCD IIIa and isolated FCD cases from the temporal and frontal lobes. Since all cases showed the same principle of preserved lamination, we hypothesize that this is a common phenomenon.

Despite the maintenance of layering, morphology of certain neuronal populations appeared severely affected: In layer III we either observed a rarefication of SMI32-positive pyramidal cells and/or a shortening of their dendrites. Since dendritic differentiation of pyramidal cells occurs when migration is completed and they have reached their final positions, this pathological pattern indicates a late developmental defect [37]. In frontal FCD, however, we observed additional accumulations or malformations of SMI32-positive cells, indicating that this FCD group is more severely affected. Accordingly, layer IV was always preserved in temporal FCD, where SMI32-positive pyramidal cells respected the borders of layer III and layer V/VI and RORß-positive cells remained within layer IV.

These results are partly in line with [30] who described in cases with temporal sclerosis a preserved pattern of RORß-expressing cells in layer IV. The same study, reported dispersion or thinning of layer IV in patients with temporal FCD contrasting our findings. In our study, a comparable pattern was characteristic for frontal FCD where SMI32-positive pyramidal cells invaded layer IV and the dense band of RORß-positive neurons was dispersed to layer III, but not for our temporal FCD cases.

Concerning the lower neocortical layers, our investigations revealed that in some patients neither SMI32-positive pyramidal cells nor ER81-positive cells of layers V and VI respected the gray/white matter border, but were detected in the white matter. This subcortical occurrence points to a local migratory failure or to an incomplete regression of subplate neurons [38]. Interestingly, invasion of white matter was common to all different FCD types, independent of their localization and is most likely the cellular correlate of a blurred gray/white matter boundary seen in MR imaging [6].

How can these data be interpreted? The mammalian neocortex develops by radial migration of neuroblasts from the ventricular zone to the pial surface and the sequential formation of the six neocortical layers [39]. Thus, the general maintenance of six-layered cortical structure seen in our FCD cases suggests a postmigrational disturbance of development. Yet, the different severities of temporal and frontal malformations in our FCD cases point to a differential sensitivity of cortical areas. In fact, there is increasing evidence that different cortical regions represent separate developmental units orchestrated by individual master genes [40]. Thus, it is well conceivable that the temporal and frontal lobes are differentially susceptible to precipitating injuries during early brain development. Moreover, the fact that FCD IIIa is the most homogeneous group concerning layer preservation points to a lesion-induced cause.

### Parvalbumin and calbindin interneuron populations are not generally reduced in FCD

Our cell counts revealed that densities of parvalbumin- and calbindin-positive interneurons are highly variable in all FCD groups ranging from patients with strongly reduced to patients with increased numbers when compared to healthy controls. Thus it appears as if at least these two interneuron subtypes are not generally reduced in FCD contradicting the idea that epileptogenic brain regions are always characterized by loss of inhibitory interneurons. Yet, we cannot rule out functional changes in inhibitory transmission from our data.

Specifically, we found a high variability in the numbers of parvalbumin-positive interneurons in isolated FCD cases of the frontal and temporal lobe with no significant differences in the mean compared to controls in layer IV, which is the layer with the highest density of these cells. Previous studies reported a reduction of parvalbumin-positive cells in isolated temporal FCD; however in these studies either the number of patients was very small [41], or the reduction was only seen in a subset of FCD patients and was not quantified [42]. This selective reduction in only a subset of patients is comparable to our study, in which some patients with isolated FCDs also showed strongly reduced numbers of parvalbumin-positive cells. A dependency of the degree of loss of parvalbumin-positive interneurons with the degree of dysplasia has been shown for FCD I (a and b) and IIa [35], however, we did not observe such a dependency in our patient cohort and thus grouped FCD Ia, Ib and IIa. Hence within the group of mild FCDs, the grade cannot account for the large variability that we observed in our patients with isolated FCDs. To ensure that limitation of quantification to layer IV did not mask any significant results we quantified parvalbumin mRNA expression by RT-PCR with mRNA extracted from all cortical layers. This analysis showed a trend to reduced parvalbumin mRNA expression in all FCD cases, however, the results did not reach significance, again most likely due to high variability across patients. In contrast, our patient group with FCD IIIa differed from isolated FCDs since the variability in numbers of parvalbumin-positive cells was much smaller and the reduction of these cells reached significance compared to controls, in line with previous reports [35,43]. Again, our data indicate that FCD IIIa represents a more homogeneous entity, also with respect to interneurons.

Furthermore, our study showed a noticeable pattern of calbindin-positive cells: Besides a highly variable distribution in all groups except controls, increased mean numbers of calbindin-positive cells were observed in patients with AHS, independent of whether AHS was combined with FCD (FCD IIIa) or without. A recent report [43] found that the number of calbindin-positive cells in FCD IIIa were comparable to controls and not generally reduced; however, an increase in the number of calbindin-positive interneurons was not reported. Yet, the high variability in numbers of calbindin-positive cells in our study indicates that, again, patient selection might explain the different results. Interestingly, the numbers of calbindin-positive cells in frontal FCD were not significantly different from controls; a result which is in contrast to a study [31], which showed a general reduction of calbindin- and parvalbumin-positive cells in frontal FCD. However, in this paper, only FCD IIa and FCD IIb cases were investigated and so the strong loss of inhibitory neurons might be due to the more severe pathology, since our patient cohort included only FCD Ia, Ib and IIa cases.

In summary, our data on interneurons show that in contrast to controls, which always showed very homogeneous cell numbers, interneurons in the dysplastic cortex are differentially affected but that neither clinical patterns, nor the type or localization of FCD allow to draw direct conclusions on the loss or survival of interneurons.

### Comparison with etiology

We aimed at correlating clinical parameters with changes in cell numbers, however, this analysis revealed that clinical parameters strongly depend on the particular FCD pathology and the age of the patient. We found a correlation between seizure frequency (daily or monthly) and the ratio of TLE4/NeuN-positive cells; yet, the majority of patients with monthly seizures belonged to FCD IIIa which has a lower ratio of these cells. Similarly, the correlation between number of seizures as well as age at surgery with the number of calbindin-positive cells was strongly dependent on the FCD type, as patients with isolated FCD underwent surgery earlier in life than patients with FCD IIIa. Thus, we cannot conclude from this correlation that the number of TLE4-positive or calbindin-positive cells might have an effect on the severity of the disease. Yet, our data confirm the notion that the age of the patient has a strong influence on seizure frequency [44]. In addition, our data are in agreement with a report [45] which showed that patients with isolated FCDs (and in particular frontal FCD) have higher seizure frequencies than patients with associated AHS (FCD IIIa). Altogether, our data support the idea that FCD IIIa represents a more homogeneous entity when compared to isolated FCD cases concerning origin [18], seizure type [45] and interneuron characteristics. In addition, our data show that cellular parameters in mild isolated FCDs (Ia, Ib and IIa) are very variable across patients making it impossible to draw conclusions on structural changes to clinical parameters and vice versa.

## Conclusions

In summary, this systematic study with layer-specific markers and interneuron stainings provides a better insight in differences between isolated FCD of the temporal or frontal lobe and FCD IIIa. The basic preservation of cortical structure supports the hypothesis that late cortical development is disturbed in FCD, yet most likely with different causes depending on brain region, FCD type and FCD severity.

## Methods

### Patient selection

A total of 52 cortical specimens from FCD patients aged 1–57 years (mean + standard deviation 26 ± 17 years) with FCD Type Ia and b, IIa and IIIa; and 14 control cases without FCD (mean 44 ± 17 years, range 16–70 years) and three autopsy cases (mean age at death 68 ± 25 years, range 40–88) were included in this study. The specimens were derived from the temporal or frontal lobe and were divided into experimental subgroups. The morphological analysis included 32 patients: 7 frontal (FCD Type Ia, b and IIa), 25 temporal [9 isolated (FCD Type Ia and IIa), and 16 cases with AHS (FCD Type IIIa)]. For comparison of isolated FCD with dual pathology cases we grouped all frontal (FCx) and temporal (TCx) cases, respectively. Controls (without FCD) included 3 frontal and 7 temporal specimens and three autopsy samples (frontal and temporal lobe) (for details see Additional file 1: Table S1). The group for real time RT-PCR experiments included 20 patients: 4 frontal (FCD Type Ia and IIa), 16 temporal (3 isolated (FCD Type I, IIa) and 13 FCD IIIa) and 7 controls (1 frontal, 6 temporal) (see Additional file 1: Table S2). Clinical details of all FCD cases and controls are shown in Additional file 1: Table S1 and Additional file 1: Table S2. All tissue samples were collected between 2007 and 2011.

All FCD patients and control cases had undergone neurosurgical resections due to pharmaco-resistant epilepsy. In all patients, removal of cortical tissue was clinically warranted to achieve seizure control. Pre-surgical assessment included the documentation of a detailed history, long-term video EEG monitoring, neurological examination and neuropsychological testing. Informed consent was obtained from patients and controls according to the declaration of Helsinki. The Ethics Committee at the University Clinic Freiburg approved the selection process and procedures.

### Neuropathological examination of FCD specimens

The cortical specimens of FCD patients were classified on paraffin sections by the Department of Neuropathology of the University of Freiburg initially according to [12] and then reclassified according to [19]. In patients with additional hippocampectomy the hippocampal specimens were graded qualitatively according to [46].

### Tissue preparation

All cortical specimens were collected immediately after surgical excision. For morphological analysis, slices of about 2 mm thickness were cut in isotonic saline perpendicular to the brain surface, followed by immersion-fixation in 4% paraformaldehyde (PFA) in 0.1 M phosphate buffer (PB), pH 7.4, for 24 - 48 h at 4°C and by cryoprotection in 20% sucrose in PB overnight. Cryostat sections (50 μm, orthogonally to the cortical surface) were prepared, collected in tissue culture dishes and either rinsed in PB for immunohistochemistry or in 2 × SSC (1 × SSC = 0.15 M NaCl, 0.015 M sodium citrate, pH 7.0) for *in situ* hybridization.

For RNA preparation and PCR analysis, cortical specimens were immediately snap-frozen in liquid nitrogen and stored at −80°C until further use.

### Immunohistochemistry

Free-floating tissue sections were washed in PB and pre-treated in 10% normal serum and 0.25% Triton X-100 in PB for 30 min. The sections were incubated overnight at room temperature in the presence of 1% normal serum and 1% Triton X-100 with the following primary antibodies: mouse monoclonal anti-calbindin, mouse monoclonal anti-parvalbumin (both 1:10.000; Swant), mouse polyclonal anti-SMI32 (1:1000; Covance), mouse monoclonal anti-NeuN (1:1000; Millipore), rabbit polyclonal anti-transducin-like enhancer 4 (TLE4, 1:50; Santa Cruz Biotechnology). After washing in PB tissue sections were incubated with the secondary antibody conjugated with cyanine-2 (1:200) or cyanine-3 (1:400; Jackson Immuno Research Laboratories) for three hours at room temperature followed by several washing steps in PB for at least 1.5 hours, mounting on glass slides and coverslipping with IMMU-Mount (ThermoShandon).

### In situ hybridization

Digoxigenin (DIG)-labeled anti-sense or sense riboprobes specific for retinoid-related orphan receptor ß (RORß) or ER81 mRNAs were generated by *in vitro* transcription from *Image* clones containing a 3.5 kb human RORß cDNA (Gene bank accession number: BC0051830) or a 2.5 kb human ER81 cDNA (Gene bank accession number: BC005645) ligated into the pCMV-SPORT6 transcription vector (Invitrogen) as described previously [47].

For *in situ* hybridization (ISH) cryostat sections were pretreated in a 1:1 mixture of 2 × SSC: hybridization buffer (50% formamide, 4 × SSC, 250 g/ml heat-denatured salmon sperm DNA, 100 g/ml tRNA, 5% dextransulfate and 1% Denhardt’s solution) for 15 min and prehybridized in hybridization buffer for 60 min at 55°C. Hybridization was performed in the same buffer including DIG-labeled anti-sense or sense RORß or ER81 cRNA probes (ca. 100 ng/ml) at 55°C overnight. After hybridization, the brain sections were washed in 2 × SSC (2 × 15 min) at room temperature, 2 × SSC and 50% formamide, 0.1 × SSC and 50% formamide for 15 min at 65°C, and finally in 0.1 × SSC (2 × 15 min) at 65°C. Immunological detection of DIG-labeled hybrids was performed with an anti-DIG antibody from sheep conjugated with alkaline phosphatase as recommended by the manufacturer (Roche). Colorimetric detection was accomplished using nitroblue tetrazolium and 5-bromo-4-chloro-3-indolylphosphate. Development of the color reaction was performed in the dark and stopped by transfer into H_2_O.

### Cell counting

Counting of immunolabeled cells was performed in three consecutive sections/patient or control and for four markers (calbindin, parvalbumin, NeuN and TLE4) using the *StereoInvestigator* image analysis system (MicroBrightField). Regions of interest (ROIs) were outlined at low magnification: layer I/II for calbindin, layer IV for parvalbumin, layer III and layer V/VI for NeuN and layer V/VI for TLE4. All immunopositive cells were counted within each ROI using a 20x objective; cells crossing the borders of the ROI were not included. Cell density for each ROI was determined by calculating the number of cells/area in mm^2^ and subsequently averaged for each patient. Cells were only counted in sections with good signal to background ratio in the staining resulting in different patient numbers for some stainings.

### RNA extraction, reverse transcription and quantitative real time RT-PCR

For RNA extraction, frozen neocortical specimens were thawed on ice and dissected (removal of white matter) in preparation solution containing 70% ammonium sulphate, 25 mM sodium citrate, pH 5.2, and 10 mM EDTA. 30 mg of tissue was used for RNA preparation using the RNeasy Mini Kit (QIAGEN). RNA integrity was determined by an Agilent 2100 Bioanalyzer (Agilent Technologies) and the RNA 6000 Nano Kit (Agilent) according to the manufacturer’s instructions.

Reverse transcription was performed in the presence of 1 μg total RNA (RNA integrity values 7–10) using the Maxima First Strand cDNA synthesis Kit (Fermentas) based on random decamer and oligod(T) priming. Expression of mRNAs (see below) was quantified by real-time RT-PCR on an IQ5 Real-Time PCR Detection System (Bio-Rad Laboratories) in the presence of SYBR Green (ABgene). The following human-specific primer pairs were used at 70 nM: ER81: forward 5′-CCCTCCATCGCAGTCCATAC-3′, reverse 5′-CGTCGGCAAAGGAGGAAAG-3′; RORß: forward 5′-CAAAGCGGATAACAGGCTTCA-3′, reverse 5′-AGGCACGGCACATTCTCACT-3′; S12: forward 5′- AGTTGGTGGAGGCCCTTTGT-3′, reverse 5′-AGGCCTACCCATTCTCCTAGTTTC-3′, calbindin: forward: 5′-TTACTGAAGGATCTGTGCGAGAAG-3′, reverse: 5′-CATCCGACAAAGCCATTATGTTC-3′; parvalbumin: forward 5′-TACCGACTCCTTCGACCACAA-3′, reverse 5′-TTCTTCACATCATCCGCACTCTT-3′; TLE4: forward 5′-ATCAGCCACCCATGGCAATAA-3′, reverse 5′-CGACCATCAGGGAGCAATCT-3′. Cycling conditions were as follows: 15 min at 95°C followed by 50 cycles of 15 sec at 95°C and 1 min at 60°C. Melting curves of the amplified products were used to control for specificity of the amplification reaction. The resulting fluorescence values (obtained by the iQTM5 Optical System Software) were background subtracted for exposure time, background fluorescence, and well factors yielding individual relative fluorescence values (RFU), which represent amplification traces of every amplicon and every cycle. RFUs were corrected for differences in PCR efficiency using the LinReg programme (LinReg, Version 11.1.0.0, HRFC, Netherlands) yielding the initial cDNA concentration/sample. Finally, all values were normalized to an endogenous control (S12 RNA) to account for variability in the initial cDNA concentration.

### Statistical analyses

All statistical analyses were performed with GraphPad Prism 4 software. For multiple comparisons a one-way analysis of variance (ANOVA) followed by Tukey’s multiple comparison test was performed. Pairwise comparison for cell numbers in patients with daily or monthly seizures was performed using a Student’s t-test. Significance levels were set as follows: * p < 0.05, ** p < 0.01. To make sure that differences between isolated FCD cases and FCD IIIa do not depend on differences in group sizes, we performed a bootstrapping method and compared variances. To relate clinical findings (age at onset, age at surgery, epilepsy duration) to cell numbers, a linear regression was calculated.

## Competing interests

The authors declare that they have no competing interests.

## Authors’ contributions

Study concept and design: Carola A. Haas (CH), Susanne Fauser (SF). Acquisition of data: SF, Susanne Huber, Catharina Donkels (CD). Analysis and interpretation of data: CH, SF, CD, Ute Häussler (UH). Drafting of the manuscript: CH, SF, UH. Critical revision of the manuscript for important intellectual content: CH, SF, UH. Statistical analysis: UH, CD. Obtained funding: CH, SF. Administrative, technical, and material support: Julia Nakagawa, Marco Prinz, Andreas Schulze-Bonhage, Josef Zentner. Study supervision: SF, CH. All authors read and approved the final manuscript.

## Supplementary Material

Additional file 1: Tables S1 and S2 Clinical data of patients and controls investigated in this study. Age represents the age (in years) of the patients at surgery (dysplasia/controls from surgery) or death (autopsy). Onset refers to the age at which a first epileptic seizure was documented (in years). Duration indicates the duration of the epileptic disorder in years. Frequency indicates the frequency of seizure attacks. AHS, hippocampal sclerosis; FCD, focal cortical dysplasia; AHE, amygdalohippocampectomy; n. a., not available. Click here for file
