# Supplementary material for: Disorganization of neocortical lamination in focal cortical dysplasia is brain-region dependent: evidence from layer-specific marker expression
Source: Acta Neuropathol Commun. 2013 Aug 8;1:47. doi: 10.1186/2051-5960-1-47 (PMC3893528; doi:10.1186/2051-5960-1-47)
Supplement: Additional file 1: Tables S1 and S2 — Clinical data of patients and controls investigated in this study. Age represents the age (in years) of the patients at surgery (dysplasia/controls from surgery) or death (autopsy). Onset refers to the age at which a first epileptic seizure was documented (in years). Duration indicates the duration of the epileptic disorder in years. Frequency indicates the frequency of seizure attacks. AHS, hippocampal sclerosis; FCD, focal cortical dysplasia; AHE, amygdalohippocampectomy; n. a., not available. [file 2051-5960-1-47-S1.docx]

**Table S1**

**Clinical data of FCD cases (A), controls (B) and autopsy cases (C) used for morphological analysis**

| **A. Patients with cortical dysplasia** | | | | | | | | | | |
| --- | --- | --- | --- | --- | --- | --- | --- | --- | --- | --- |
| **Patient**  **ID** | **Lobe** | **Sex** | **AHS** | **Age at epilepsy onset (years)** | **Age at surgery**  **(years)** | **Duration of epilepsy**  **(years)** | **Seizure**  **frequency** | **Histopathology**  **FCD Type nach**  **ILAE** | **MRI findings** | **Outcome** |
| 1 | frontal | f | no | 7 | 23 | 16 | Several/day | Ia | Blurred gray-white matter transition in the right frontal lobe | Engel IIIa |
| 2 | frontal | f | no | 7 | 14 | 7 | Several/week | Ia | Cryptogenic | Engel Ia |
| 3 | frontal | m | no | 1 | 5 | 4 | 20-50/day | Ia | FCD right frontal | Engel Ia |
| 4 | frontal | f | no | 6 | 16 | 10 | Several/day | Ib | Cryptogenic | Engel IVb |
| 5 | frontal | m | no | 10 | 21 | 11 | 2-3/night | IIa | Blurred gray-white matter transition in the left frontal lobe (precentral area to the middle frontal gyrus) | Engel IIIa |
| 6 | frontal | f | no | 4 | 50 | 46 | 30/month | IIa | Left fronto-polar enlargement of the cortical ribbon, blurred gray-white matter boundary | Engel IVb |
| 7 | frontal | m | no | 2 | 41 | 39 | 1-3/hour | IIa | Transmantle dysplasia right pericentral | Engel Ia |
| 8 | temporal | m | no | 8 | 20 | 12 | Several /day | Ia | Temporo-polar gray-white matter blurring | Engel Ia |
| 9 | temporal | m | no | 1 | 3 | 2 | n.a. | Ia | Large temporo-occipital dysplasia | Engel IIIa |
| 10 | temporal | m | no | 2 | 8 | 6 | Several/week | Ia | Circumscribed FCD in the superior temporal gyrus | Engel IVb |
| 11 | temporal | m | no | 0 | 9 | 9 | Several/day | Ia | Blurring of the gray-white-matter transition temporal extending into the occipital lobe | Engel IIa |
| 12 | temporal | m | no | 0 | 2 | 2 | 10 /day | Ia | Malformation of the right hemisphere with enlarged ventricles and sulci | Engel Ia |
| **A. Patients with cortical dysplasia (continued)** | | | | | | | | | | |
| **Patient**  **ID** | **Lobe** | **Sex** | **AHS** | **Age at epilepsy onset (years)** | **Age at surgery**  **(years)** | **Duration of epilepsy**  **(years)** | **Seizure**  **frequency** | **Histopathology**  **FCD Type nach**  **ILAE** | **MRI findings** | **Outcome** |
| 13 | temporal | f | no | 0 | 8 months | 8 months | n.a. | IIa | Hemimegalencephaly | Engel Ia |
| 14 | temporal | m | no | 6 | 15 | 9 | Several/day | IIa | Cryptogenic | Engel Ia |
| 15 | temporal | m | no | 0 | 1 | max.1 year | n.a. | IIa | Dysplasia temporo-parieto-occipital | Engel Ia |
| 16 | temporal | f | no | 4 | 10 | 6 | Several /month | IIa | FCD right mesio-occiptal | Engel IVb |
| 17 | temporal | f | yes | 0 | 27 | 27 | Several /month | IIIa | AHS and temporo-polar gray-white matter blurring | Engel IIa |
| 18 | temporal | m | yes | 3 | 37 | 34 | 10 /month | IIIa | AHS and temporo-polar gray-white matter blurring | Engel Ia |
| 19 | temporal | m | yes | 1 | 49 | 48 | 10-15 /month | IIIa | AHS and temporo-polar gray-white matter blurring | Engel Ia |
| 20 | temporal | f | yes | 5 | 8 | 3 | Several /month | IIIa | AHS and temporo-polar gray-white matter blurring | Engel Ia |
| 21 | temporal | f | yes | 0 | 31 | 31 | 4-6 /month | IIIa | AHS and temporo-polar gray-white matter blurring | Engel Ia |
| 22 | temporal | m | yes | 6 | 14 | 8 | Several /week | IIIa | AHS and temporo-polar gray-white matter blurring | Engel Ia |
| 23 | temporal | m | yes | 6 | 17 | 11 | 1-2 /month | IIIa | AHS in MRI | Engel Ia |
| 24 | temporal | m | yes | 35 | 47 | 12 | 13-18 /month | IIIa | AHS and temporo-polar gray-white matter blurring | Engel Ia |
| 25 | temporal | m | yes | since childhood | 55 | > 40 years | 1 /week | IIIa | AHS and temporo-polar gray-white matter blurring | Engel IIb |
| 26 | temporal | m | yes | 1 | 39 | 38 | Several/month | IIIa | AHS and temporo-polar gray-white matter blurring | Engel Ia |
| 27 | temporal | f | yes | 2 | 25 | 23 | Several series /week | IIIa | AHS and right hemispheral atrophy | Engel Ia |
| 28 | temporal | m | yes | 0 | 35 | 35 | Several/month | IIIa | AHS and temporo-polar gray-white matter blurring | Engel Vb |
| **A. Patients with cortical dysplasia (continued)** | | | | | | | | | | |
| **Patient**  **ID** | **Lobe** | **Sex** | **AHS** | **Age at epilepsy onset (years)** | **Age at surgery**  **(years)** | **Duration of epilepsy**  **(years)** | **Seizure**  **frequency** | **Histopathology**  **FCD Type nach**  **ILAE** | **MRI findings** | **Outcome** |
| 29 | temporal | m | yes | 3 | 39 | 36 | Several/month | IIIa | Partial AHE 2000, dysplasia in the temporal pole | Engel II |
| 30 | temporal | f | yes | 44 | 52 | 8 | Several/month | IIIa | AHS and atrophy of the temporal pole | Engel Ia |
| 31 | temporal | m | yes | 12 | 41 | 29 | Several/month | IIIa | AHS and temporo-polar gray-white matter blurring | Engel Ia |
| 32 | temporal | m | yes | 3 | 57 | 54 | n.a. | IIIa | AHS and temporo-polar gray-white matter blurring | Engel Ia |

| **B. Control patients without cortical dysplasia** | | | | | | | | | |
| --- | --- | --- | --- | --- | --- | --- | --- | --- | --- |
| **Control**  **ID** | **Lobe** | **Sex** | **AHS** | **Age at epilepsy onset** | **Age at surgery** | **Duration of epilepsy** | **Seizure frequency** | **MRI findings** | **Outcome** |
| 1 | frontal | m | no | 24 | 54 | 30 | Several /month | Parenchymal defect left frontal following a brain trauma | Engel Ia |
| 2 | frontal | m | no | 14 | 50 | 36 | Several/month | Parenchymal defect left frontal following a brain abcess | Engel III |
| 3 | frontal | m | no | < 10 | 48 | >38 | Several/month | Cavernoma left inferior frontal gyrus, left precentral, left temporo-occipital, right frontal, right temporal | n.a. |
| 4 | temporal | m | no | 18 | 24 | 6 | Several/ month | Encephalocele left temporal | Engel Ib |
| 5 | temporal | f | no | 33 | 39 | 6 | Several/ month | Encephalocele left temporal | Engel Ia |
| 6 | temporal | m | no | 69 | 70 | 1 | Several/ year | Aneurysm of the left medial cerebral artery | Engel III |
| 7 | temporal | f | yes | 32 | 41 | 9 | Several /week | AHS and residual right temporal intracerebral hemorrhage | Engel IIb |
| 8 | temporal | f | yes | 26 | 42 | 16 | n.a. | Parenchymal defect left temporal following a brain trauma | n.a. |
| 9 | temporal | f | yes | 5 | 22 | 17 | Several/ day | AHS and left temporal cavernoma | Engel IIb |
| 10 | temporal | m | yes | n.a. | 53 | n.a. | n. a. | n.a. | n.a. |

| **C. Autopsy cases** | | | | | |
| --- | --- | --- | --- | --- | --- |
| **Control**  **ID** | **Lobe** | **Age at death** | **Profile** | **Cause of death** | **Medical history** |
| 1 | frontal and temporal | 88 | Profile A | Respiratory insufficiency with chronic obstructive pumonal disease | Latent myocardial infarction, no brain disease |
| 2 | frontal and temporal | 75 | Profile A | Polytrauma following a traffic accident | Mild general arteriosclerosis |
| 3 | frontal | 40 | Profile A | Intoxication with opiates and alcohol | No medical history |

**Table S2**

**Clinical data of FCD cases (A) and controls (B) used for real time RT-PCR**

| **A. Patients with cortical dysplasia** | | | | | | | | | | |
| --- | --- | --- | --- | --- | --- | --- | --- | --- | --- | --- |
| **Patient**  **ID** | **Lobe** | **Sex** | **AHS** | **Age at epilepsy onset**  **(years)** | **Age at surgery**  **(years)** | **Duration of epilepsy**  **(years)** | **Seizure frequency** | **Histopathology**  **Blümcke et al. 2011** | **MRI findings** | **Outcome** |
| 1 | frontal | f | no | 10th day after birth | 22 | 22 | n.a. | Ia | FCD, frontal-temporal, right | Engel Ia |
| 2 | frontal | f | no | 3 month after birth | 1 | 1 | 5-6/day | Ia | FCD, left | n.a. |
| 3 | frontal | f | no | 4 | 30 | 26 | 3-4/week | IIa | Cryptogenic | Engel Ia |
| 4 | frontal | f | no | 1 | 16 | 16 | 1-2/week | IIa | FCD, frontal, right | Engel IIIa |
| 5 | temporal | f | no | 31 | 37 | 6 | 17-19/month | Ib | Hippocampal malrotation, left  FCD, temporo-mesial and temporo-polar, left | Engel IVb |
| 6 | temporal | m | no | 1 | 36 | 36 | 18-20/month; | Ib | Cryptogenic | Engel IIIa |
| 7 | temporal | m | no | 42 | 43 | 6 months | 6-7/month | Ib | AHS, right  FCD, temporo-polar, right | Engel Ia |
| 8 | temporal | f | yes | 1 | 10 | 9 | 2-14/day | IIIa  AHS Wyler I | FCD, right | n.a. |
| 9 | temporal | m | yes | 51 | 57 | 6 | n.a. | IIIa  AHS Wyler III | AHS, right  White-gray matter blurring, temporal pole, right; FCD, right | n.a. |
| 10 | temporal | m | yes | 2 | 10 | 8 | 1-3/month | IIIa  AHS Wyler II-III | Cortex thickening temporo-polar and temporo-lateral, left | Engel IIa |
| 11 | temporal | m | yes | 2 | 36 | 34 | 2/ month | IIIa  AHS Wyler I | FCD, temporal pole, left | Engel Ia |
|  | | | | | | | | | | |
| **A. Patients with cortical dysplasia (continued)** | | | | | | | | | | |
| **Patient**  **ID** | **Lobe** | **Sex** | **AHS** | **Age at epilepsy onset**  **(years)** | **Age at surgery**  **(years)** | **Duration of epilepsy**  **(years)** | **Seizure frequency** | **Histopathology**  **Blümcke et al. 2011** | **MRI findings** | **Outcome** |
| 12 | temporal | m | yes | 27 | 36 | 9 | 1-3/week | IIIa and IIIb  AHS Wyler II | Expanding lesion, temporo-lateral and amygdala, right  White-gray matter blurring, temporal, right | Engel Ia |
| 13 | temporal | f | yes | 4 | 18 | 14 | frequently per day | IIIa  AHS Wyler III | AHS right,  bordered FCD rostral | Engel Ia |
| 14 | temporal | f | yes | 3 | 6 | 3 | 2/day | IIIa  AHS Wyler IV | Defect in right hemisphere after perinatal media infarction,  AHS, right | Engel Ia |
| 15 | temporal | m | yes | 26 | 36 | 10 | 2-3/week | IIIa  AHS Wyler I | Hyperintense lesion in amygdala and hippocampus, right | n.k. |
| 16 | temporal | m | yes | since childhood | 48 | n.a. | 4-7/month | IIIa  AHS Wyler n.a. | AHS, left  FCD, temporo-polar, left | n.a. |
| 17 | temporal | f | yes | 7 | 30 | 23 | 4/ week | IIIa  AHS Wyler n.a. | FCD, temporal pole, left  abnormal hippocampus formation, left | n.a. |
| 18 | temporal | f | yes | 10 | 22 | 12 | 5-6/month | IIIa  AHS Wyler IV | AHS left,  Meningoencephalocele at temporal pole | Engel Ia |
| 19 | temporal | f | yes | 2 | 46 | 44 | n.a. | IIIa  AHS Wyler IV | AHS left,  Lesion at gyrus frontalis medius  FCD temporal pole, left | n.a. |
| 20 | temporal | f | yes | n.k. | 2 | n.k. | 20/month during night | IIIa  AHS Wyler I | FCD, left | Engel Ia |

| **B. Control patients without cortical dysplasia** | | | | | | | | | |
| --- | --- | --- | --- | --- | --- | --- | --- | --- | --- |
| **Control**  **ID** | **Lobe** | **Sex** | **AHS** | **Age at epilepsy onset** | **Age at surgery** | **Duration of epilepsy** | **Seizure frequency** | **MRI findings** | **Outcome** |
| 1 | frontal | m | no | n.a. | 48 | n.a. | n.a. | Cavernoma left inferior frontal gyrus, left precentral, left temporo-occipital, right frontal, right temporal | n.a. |
| 2 | temporal | m | no | n.a. | 69 | n.a. | 2 noticed seizures during this period | Aneurysm of the left medial cerebral artery | Engel III |
| 3 | temporal | m | no | 17 | 18 | 1 | 2/month | Dysembryoplastic neuroepithelial tumour (DNET)grade I, temporal, right | Engel Ia |
| 4 | temporal | m | no | 12 | 18 | 6 | 15/month | Gangliocytoma grade I, temporo-mesial, right | Engel Ia |
| 5 | temporal | f | yes | 5 | 21 | 16 | 6 month | AHS and left temporal cavernoma | Engel IIb |
| 6 | temporal | f | yes | 10 | 27 | 17 | 2/ year | AHS, left  FCD gyrus temporalis superior, left | Engel IIIa |
| 7 | temporal | f | yes | 8 | 16 | 8 | 3/ week | Ganglioglioma grade I temporal, right (resection 7 years ago) | Engel Ia |
